# Optimizing Voice Sample Quantity and Recording Settings for the Prediction of Type 2 Diabetes Mellitus: Retrospective Study

**DOI:** 10.2196/64357

**Published:** 2025-06-26

**Authors:** Atousa Assadi, Jessica Oreskovic, Jaycee Kaufman, Yan Fossat

**Affiliations:** 1 Klick Applied Sciences, 175 Bloor St East, North Tower, 3rd floor, Toronto, ON, M4W3R8, Canada, 1 6472068717

**Keywords:** vocal biomarker, acoustic biomarker, voice analysis, type 2 diabetes, diagnostics, digital phenotyping, voice data

## Abstract

**Background:**

The use of acoustic biomarkers derived from speech signals is a promising non-invasive technique for diagnosing type 2 diabetes mellitus (T2DM). Despite its potential, there remains a critical gap in knowledge regarding the optimal number of voice recordings and recording schedule necessary to achieve effective diagnostic accuracy.

**Objective:**

This study aimed to determine the optimal number of voice samples and the ideal recording schedule (frequency and timing), required to maintain the T2DM diagnostic efficacy while reducing patient burden.

**Methods:**

We analyzed voice recordings from 78 adults (22 women), including 39 individuals diagnosed with T2DM. Participants had a mean (SD) age of 45.26 (10.63) years and mean (SD) BMI of 28.07 (4.59) kg/m². In total, 5035 voice recordings were collected, with a mean (SD) of 4.91 (1.45) recordings per day; higher adherence was observed among women (5.13 [1.38] vs 4.82 [1.46] in men). We evaluated the diagnostic accuracy of a previously developed voice-based model under different recording conditions. Segmented linear regression analysis was used to assess model accuracy across varying numbers of voice recordings, and the Kendall tau correlation was used to measure the relationship between recording settings and accuracy. A significance threshold of *P*<.05 was applied.

**Results:**

Our results showed that including up to 6 voice recordings notably improved the model accuracy for T2DM compared to using only one recording, with accuracy increasing from 59.61 to 65.02 for men and from 65.55 to 69.43 for women. Additionally, the day on which voice recordings were collected did not significantly affect model accuracy (*P*>.05). However, adhering to recording within a single day demonstrated higher accuracy, with accuracy of 73.95% for women and 85.48% for men when all recordings were from the first and second days.

**Conclusions:**

This study underscores the optimal voice recording settings to reduce patient burden while maintaining diagnostic efficacy.

## Introduction

Diabetes mellitus is a chronic metabolic disorder characterized by persistent elevated blood glucose levels due to inadequate or impaired insulin production or utilization. It affects 10.5% of the worldwide population (536.6 million people) [1], with type 2 diabetes mellitus (T2DM) accounting for 90% of cases [2]. Uncontrolled diabetes is a major contributors to cardiovascular disorders, blindness, renal failure, and lower limb amputation [2].

Traditional diagnostic methods of fasting plasma glucose and oral glucose tolerance tests involve blood sampling, which can cause inconvenience or discomfort to patients owing to frequent monitoring. Moreover, the lack of a glucometer and the time spent for self-testing are barriers in the self-management of diabetes [3,4]. In response to these challenges, acoustic biomarkers from speech signals have emerged as promising non-invasive alternatives, offering a convenient solution for diagnosing and monitoring diabetes, especially for individuals in remote areas with restricted health care accessibility.

Sustained periods of high blood glucose and the detrimental effects of peripheral neuropathy and myopathy in individuals with T2DM impact the elastic properties of the vocal folds [5], weaken the laryngeal muscles, and induce respiratory changes [6]. These physiological changes can affect voice parameters, leading to voice disorders like hoarseness [7] and dysphagia [8]. Consequently, compared to those without T2DM, individuals with the condition exhibit significant vocal differences, quantified by phonation time, fundamental frequency, jitters, and shimmers [6], highlighting the importance of investigating vocal variations as potential markers of T2DM [9-13].

Our group previously assessed the feasibility of using voice recordings from mobile applications to detect T2DM [14]. Our results demonstrated that voice biomarkers—specifically pitch, jitters, and shimmers—combined with age and BMI could predict T2DM with an accuracy of 0.89 for women and 0.86 for men [14]. However, requiring participants to record their voices at least 6 times daily over a 2-week period posed challenges related to participant burden and recording consistency.

Therefore, this study aims to optimize the voice sampling process by determining (1) the minimum number of voice samples required, and (2) the optimal recording schedule (frequency and timing) needed to maintain diagnostic accuracy while reducing participant burden. We hypothesize that a more streamlined voice sampling protocol can achieve comparable predictive performance to prior studies while improving feasibility for long-term diabetes monitoring.

## Methods

### Study Design

To address the objectives of this project, we designed a retrospective study based on our previously developed model and the same dataset that yielded the highest balanced accuracy [14]. The original data collection took place between August 30, 2021, and June 30, 2022 in India [14]. In total, 505 participants (mean [SD] age 41.03 [13.29] years, 336 male participants) were recruited and instructed to record a short English phrase (“Hello. How are you? What is my glucose level right now?”) up to 6 times daily using their smartphone for 14 consecutive days.

### Participants and Measurements

A balanced subset of the original dataset was used for this analysis and included 78 participants (aged >18 years old, 22 women), with 39 diagnosed with T2DM [14]. Participants in the T2DM and non-T2DM groups were matched for age and BMI to minimize the demographic impact on voice recordings (Table 1). A T2DM diagnosis was confirmed by a physician according to the American Diabetes Association guidelines [15]. All participants were nonsmokers, had no diagnosed neurological or speech impairments, and signed the consent forms.

**Table 1.** Patient demographic characteristics.

Variable	Total	Non-T2DM^a^ group	T2DM group
N (%)	78 (100.0)	39 (50.0)	39 (50.0)
Women	22 (28.21)	11 (50.0)	11 (50.0)
Men	56 (71.79)	28 (50.0)	28 (50.0)
Age (years), mean (SD)	45.26 (10.63)	45.49 (10.8)	45.03 (10.58)
Women	45.82 (10.4)	45.91 (10.85)	45.73 (10.47)
Men	45.04 (10.8)	45.32 (10.98)	44.75 (10.8)
BMI (kg/m^2^), mean (SD)	28.07 (4.59)	28.77 (5.01)	27.36 (4.06)
Women	30.25 (5.35)	31.41 (5.4)	29.09 (5.29)
Men	27.21 (3.98)	27.74 (4.53)	26.68 (3.34)

a T2DM: type 2 diabetes mellitus.

As part of the study protocol, participants recorded their voice at least 6 times per day over a 2-week period using a custom mobile application installed on their personal cell phones. These recordings took place either at home or in a quiet environment with minimal background noise [12]. To establish a consistent starting point, a participant’s first day (d01) was defined as the day they recorded at least 2 voice samples. Voice samples recorded prior to d01 were excluded from the analysis.

### Optimizing Voice Recording Quantity and Settings for Enhanced Model Accuracy

To analyze the collected voice recordings, 14 acoustic features were extracted to characterize key parameters related to pitch, intensity, harmonic noise ratio, shimmers, and jitters [14]. Features that were significantly different between the T2DM and non-T2DM groups (*P*<.05, Cohen *d* >0.02) were included in he model development pipeline, with separate models for women and men. For women, the key features were pitch SD, mean pitch, RAP jitter, and apq3 shimmer, while for men, mean intensity, apq11 shimmer, intensity SD, and ppq5 jitter were used. A 5-fold cross-validation was performed for feature selection, threshold determination, and model optimization based on the best predictive balanced accuracy [14]. The optimal model for women was a logistic regression model (threshold of 0.3) with BMI and 3 voice features: mean pitch, pitch SD, and RAP jitter. For men, the optimal model was a naive Bayes classifier (threshold of 0.215) with age, BMI, and 2 voice features: mean intensity and apq11 shimmer.

The analysis pipeline included (1) indicating the optimal number of voice recordings for effective T2DM classification based on changes in model accuracy across varying quantities of voice samples, and (2) studying the effect of voice recording configurations on predictive performance (Multimedia Appendix 1).

To study the changes in the model’s accuracy trend across varying number of voice samples, we employed segmented linear regression by fitting two distinct linear models to the data before and after the n voice samples breakpoint. The Kendall tau measure of correlation was used to investigate the strength and direction of the relationship between ordinal variables (such as days) and model accuracy. *P* values of .05 were considered statistically significant.

### Ethical Considerations

The protocol (ID MGCTS107) received ethics approval by Saanvi Ethical Research LLP (No. MGCTS/20/107 V01), all participants provided informed consent, and data were stored in a secure cloud database with no identifying information. Participants were compensated for their time; each participant received US $56.

## Results

### Participants and Measurements

**Figure 1.**
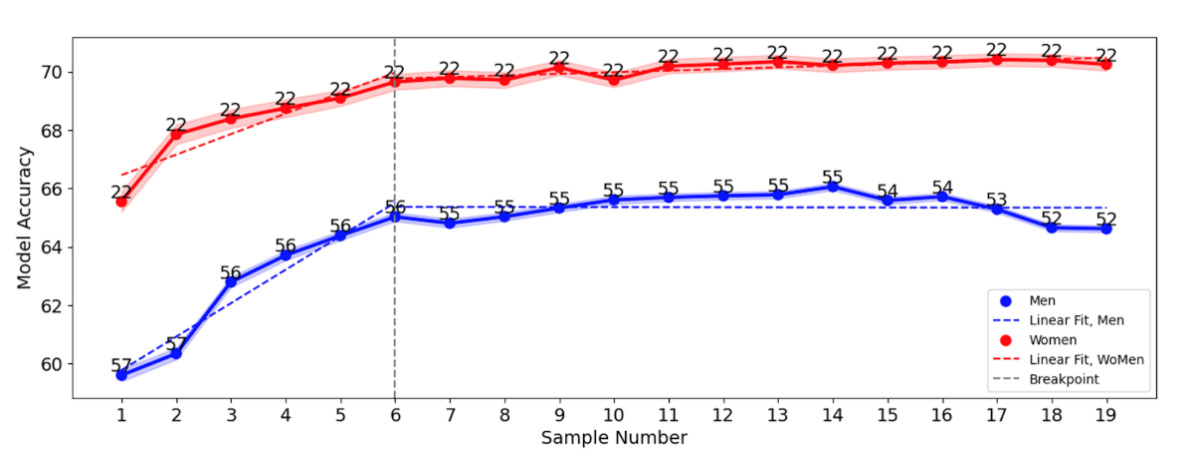
The accuracy of the model using different number of voice recordings. The lines present the average accuracy for men (blue) and women (red). The shaded area shows the confidence interval. The numbers in the figure show the number of participants whose data were included in the analysis per day.

The mean (SD) age and mean (SD) BMI of participants were 45.26 (10.63) years and 28.07 (4.59) kg/m^2^, respectively (Table 1). In total, 5035 recordings were included in the analysis, and 2620 from participants with T2DM (Table 2). The mean (SD) number of daily recordings for all participants was 4.91 (1.45) with women more adherent to the protocol than men (5.13 [1.38] vs 4.82 [1.46], Multimedia Appendix 2, Figure 1).

**Table 2.** Voice recording data.

Variable	Total	Non-T2DM^a^ group	T2DM group
N (%)	5035 (100)	2415 (48)	2620 (52)
Women, n (%)	1539 (30.6)	713 (46.3)	826 (53.7)
Men, n (%)	3496 (69.4)	1702 (48.7)	1794 (51.3)
Number of recordings per participant, mean (SD)	64.55 (19.53)	61.92 (21.67)	67.18 (17)
Women	69.95 (16.26)	64.82 (19.12)	75.09 (11.45)
Men	62.43 (20.42)	60.79 (22.82)	64.07 (17.97)

a T2DM: type 2 diabetes mellitus.

### Optimizing Voice Recording Quantity and Settings for Enhanced Model Accuracy

Both in men and women, the model accuracy improved with the inclusion of up to 6 voice samples, after which it stabilized with no significant improvement (Figure 1). The changes in the slope of the linear fit were −1.15 for men and −0.65 for women, indicating a faster accuracy improvement in men than in women with the addition of initial voice samples.

**Figure 2.**
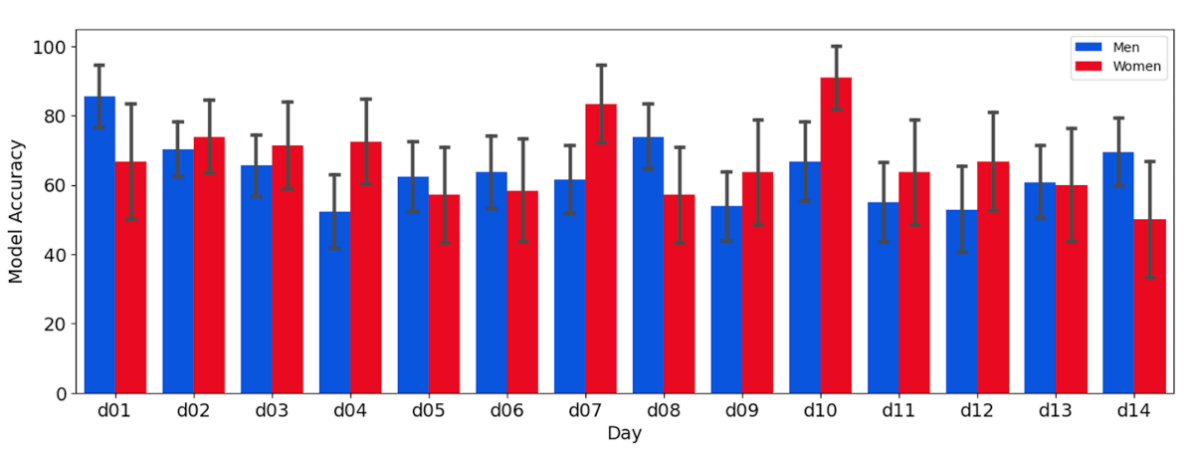
The accuracy of the model using 6 voice recordings per day. d: day.

Considering 6 voice samples for effective T2DM diagnosis, the highest model accuracy was achieved with recordings from day 1 in men, while for women, the peak accuracy was observed with recordings from day 10 (Figure 2). However, the variations in model accuracy across different days were not significant, and no statistically discernible trend was observed (*P*=.23 for men, *P*=.27 for women). The model accuracy was generally higher for women than for men on most days, although the difference was not statistically significant, as indicated by the overlapping confidence intervals.

**Figure 3.**
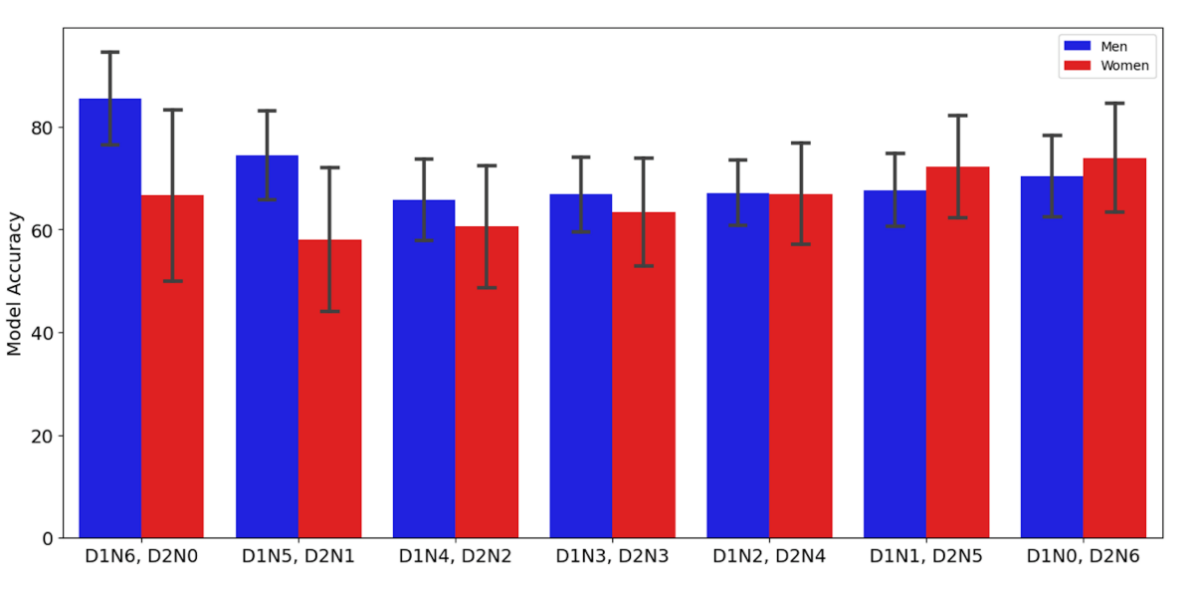
The accuracy of the model using total 6 voice recordings from day 1 and day 2. D1: day 1, D2: day 2, N: number of samples from the day.

Moreover, we observed that the model accuracy was higher in men than in women when the majority of recordings were from the first day (Figure 3). As the distribution of recordings were balanced between the first and second days, the accuracy gap between men and women narrowed. Finally, when the majority of recordings were from the second day, the model accuracy was slightly higher for women than for men, with the differences in accuracy levels becoming less pronounced. Our statistical test indicated no significant trend in the model accuracy for men when using 6 recordings across 2 days (*P*>.99). For women, there was a significant increasing trend in the model accuracy when the majority of recordings were taken on the second day (*P*=.03), suggesting that consistent participation in women can improve the model performance.

## Discussion

### Principal Findings

This research, to our knowledge, is the first to investigate the optimal balance between the number and settings of voice recordings for effective T2DM diagnosis, with the goal of reducing patient burden. Our findings indicated that 6 voice recordings are sufficient to maintain diagnostic accuracy, improving patient compliance and accessibility for T2DM screening. No significant differences in model accuracy were observed across different days while adherence to recording in a single day showed higher accuracy. This study lays the groundwork for future research and clinical applications focused on optimizing health care delivery for T2DM.

### Comparison to Prior Works

Previous studies have shown that an increased burden from the treatment and self-management of chronic health conditions such as T2DM is associated with higher levels of distress, lower adherence to self-care routines, decreased satisfaction with medications, reduced quality of life, poorer physical and mental health, and greater risk of complications and deaths [4,16-18]. In alignment with these, our study demonstrated that up to 6 voice recordings are sufficient to effectively diagnose T2DM, thereby reducing patient burden while maintaining diagnostic accuracy.

There are conflicting reports on self-management among men and women. While Zhou et al [19] observed that women exhibited greater compliance in self-care than men did, Mogre et al [20] reported higher self-monitoring of blood glucose in men. In our study, despite the lower number of women participants than men, women showed a higher adherence to the voice recording protocol. This higher adherence among women may explain the observed increase in model accuracy, as recordings were distributed across 2 days, suggesting that consistent participation enhances the model performance.

Prior research has reported no significant day-to-day variability in voice recordings while there exists a significant time-of-day influence on acoustics with voice quality enhanced with increased voice use [21,22]. In alignment with these findings, our results showed that both in men and in women, the model accuracy was not significantly different between days while there was an increase in accuracy when the majority of the recordings were from a single day. Due to the limited distribution of samples across different times of the day, we were unable to assess the time-of-day variability.

### Strengths and Limitations

This study provides important insights into optimizing voice-based T2DM diagnostics while minimizing participant burden. However, several limitations should be considered. First, there was a limited sample size of women. The smaller number of women participants may reduce the generalizability of our findings, particularly regarding sex-specific effects. Future studies with larger, more balanced datasets are needed to validate these observations. Second, our relatively small dataset limited the use of more advanced machine learning techniques, such as neural networks. While these models may offer further improvements in the diagnostic accuracy and insight into optimal data collection strategies, future studies with larger datasets are required to fully explore their potential. Third, due to uneven distribution of recordings across different times of the day, we could not assess how the time-of-day influences voice-based diagnostics. Future studies should implement controlled recording schedules to systematically examine these effects.

## Supplementary material

10.2196/64357 Multimedia Appendix 1 Methods for optimizing voice recording quantity and settings for enhanced model accuracy.

10.2196/64357 Multimedia Appendix 2 Number of daily voice recordings per participant.

## Abbreviations

T2DM: type 2 diabetes mellitus
