# Supplementary material for: Optimizing Voice Sample Quantity and Recording Settings for the Prediction of Type 2 Diabetes Mellitus: Retrospective Study
Source: JMIR Biomed Eng. 2025 Jun 26;10:e64357. doi: 10.2196/64357 (PMC12226960; doi:10.2196/64357)
Supplement: Multimedia Appendix 1 [file biomedeng-v10-e64357-s001.docx]

# Multimedia Appendix 1: Methods

## **Optimizing Voice Recording Quantity and Settings for Enhanced Model Accuracy**

Using the developed models [14], the average T2DM probability was calculated over 5-fold cross validation for each recording. Given multiple voice recordings, a participant was classified as having T2DM if the average estimated probability equaled or exceeded the threshold. Model accuracy was determined by the number of correct classifications.

To indicate the optimal number of voice recordings to achieve effective T2DM diagnostic, participants with at least n voice recordings collected over study period were selected. Subsequently, for each participant, n recordings were randomly selected to indicate T2DM classification, based on the average estimated probability, and assess model accuracy. This process was repeated 2000 times to capture a wide range of combinations of voice samples per participant.

To examine the variability of diagnostic accuracy across different days using n voice recordings, we predicted T2DM for each combination of n voice recordings from participants who provided at least n voice samples per day. The accuracy of the model for each participant was estimated based on the number of correct predictions across all combinations of n voice recordings per day. The overall accuracy was estimated as the average accuracy of the model for all participants.

Finally, to investigate the effect of distributing voice recordings across two days, we calculated model accuracy in participants who have at least n1 voice samples in day 1 and n-n1 voice samples in day 2. For each participant, we calculated the average model probability given all combinations of voice recordings across 2 days. The accuracy of the model was estimated based on the number of correct predictions for each participant. The overall accuracy was estimated as the average accuracy of the model over all participants.
