# Supplementary material for: Optimizing Voice Sample Quantity and Recording Settings for the Prediction of Type 2 Diabetes Mellitus: Retrospective Study
Source: JMIR Biomed Eng. 2025 Jun 26;10:e64357. doi: 10.2196/64357 (PMC12226960; doi:10.2196/64357)
Supplement: Multimedia Appendix 2 [file biomedeng-v10-e64357-s002.docx]

# Multimedia Appendix 2: Voice Recording Information

| 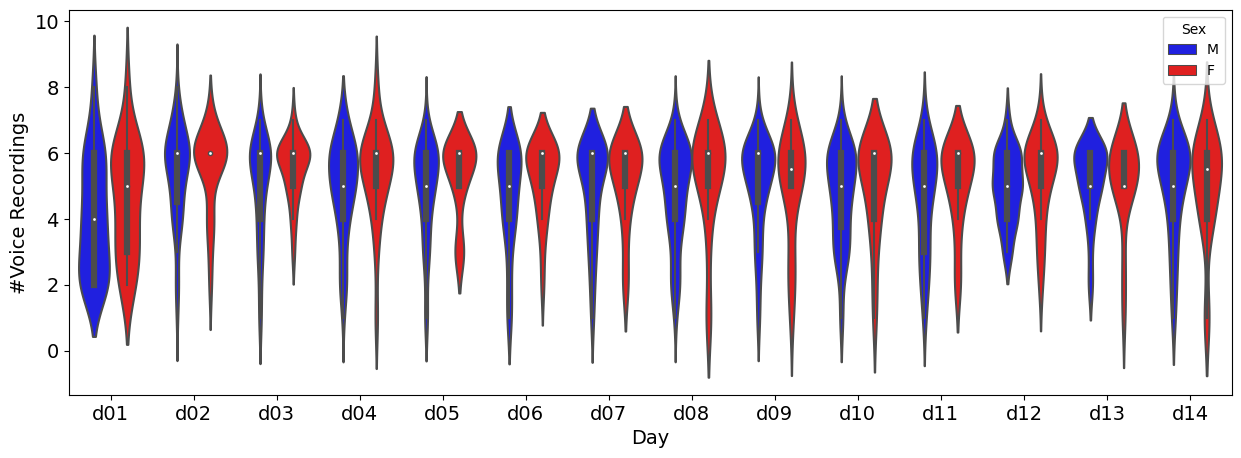 |
| --- |
| Figure 1: Number of daily voice recordings per participant. d: day. |
